# Complete mitochondrial genome of a cave dwelling *Desmopachria* (Insecta: Coleoptera: Dytiscidae) from the Eastern Amazon

**DOI:** 10.1080/23802359.2020.1870885

**Published:** 2021-02-09

**Authors:** Santelmo Vasconcelos, Renato R. M. Oliveira, Eder S. Pires, Thadeu Pietrobon, Xavier Prous, Angélico Asenjo, Guilherme Oliveira

**Affiliations:** a Instituto Tecnológico Vale, Belém, Brazil; b Vale Speleology, Avenida Dr. Marco Paulo Simon Jardim 3580, Prédio 1, Mina de Águas Claras, Nova Lima, Brazil

**Keywords:** Cave biodiversity, diving beetle, Hydroporinae, Serra dos Carajás

## Abstract

Coleoptera presents most of the cave fauna biodiversity, with several troglobite species belonging to the aquatic family Dytiscidae. However, very little is known on both genetic and genomic diversity traits of Neotropical cave beetles. Thus, here we present the complete mitochondrial genome sequence of five specimens of *Desmopachria* collected in a ferruginous cave from Serra dos Carajás in Parauapebas (Pará, Brazil, Eastern Amazon). Besides the general characteristics of the mitogenome of the analyzed specimens, we present their phylogenetic position within the family, considering the available genome sequences of different subfamilies within Dytiscidae.

Coleoptera, the most diverse animal order, also presents most of the known troglobitic diversity, with approximately 2000 known species from cave environments (Moldovan 2005). However, there is still very little information on Neotropical cave beetles, especially regarding genetic and genomic traits. Several species of Dytiscidae, the predaceous diving beetle family, present specializations to the troglobitic habit (Moldovan 2005). In Brazil, the family is exceptionally diverse (Nilsson and Hájek 2018), with many species yet to be formally described. *Desmopachria* is a genus of small beetles (generally smaller than 2.5 mm) with ca. 130 species from southern Nearctic and Neotropical regions, belonging to tribe Hyphydrini of the subfamily Hydroporinae, which consists of 10 tribes, 117 genera and approximately 2300 species (Braga and Ferreira Jr. 2018, Nilsson and Hájek 2018). In this context, we describe the mitogenome of five specimens of *Desmopachria* sp. collected in the cave N5S_0010 in Serra dos Carajás (6°06′20.5”S, 50°07′53.0”W), Parauapebas, Pará, Brazil (Eastern Amazon).

Total genomic DNA was isolated from the samples using the DNeasy Blood & Tissue Kit (Qiagen), following the manufacturer’s protocol for insects. The samples were deposited at Instituto Tecnológico Vale (ITV) under the accession numbers ITV986, ITV9391, ITV9392, ITV9393 and ITV9395. Paired-end libraries were constructed from ∼50 mg of DNA using the QXT SureSelect (Agilent Technologies) kit and the sequencing run was performed in the Illumina NextSeq 500 platform using the high-output v2 kit (300 cycles). Mitochondrial genome assemblies were performed with NovoPlasty 3.6 (Dierckxsens et al. 2017) and the genes were annotated with MITOS2 (Bernt et al. 2013), with minor manual corrections in Artemis v17 (Carver et al. 2012) and Geneious Prime 2020.2 (Biomatters). All available complete mitogenomes of Dytiscidae species were recovered from GenBank, with species of Carabidae, Gyrinidae and Haliplidae as outgroups, and all protein sequences were aligned with MAFFT v7.271 (Katoh et al. 2002). For the phylogenetic analyses, we used maximum likelihood (ML) and Bayesian inference (BI) with RAxML v8.2 (Stamatakis 2014) and MrBayes v3.2.6 (Ronquist et al. 2012), respectively, as implemented in the CIPRES portal (http://www.phylo.org) with the parameters employed by Vasconcelos et al. (2018).

The complete mitochondrial genomes of *Desmopachria* sp. (GenBank accessions MH643816, MW007723, MW007724, MW007725 and MW007726), which are the first for members of the tribe Hyphydrini (Hidroporinae), presented a small variation in sizes, ranging between 16,583 and 16,616 pb, due to two indels in the control region, the first one consisting of the addition of the dinucleotide AT in ITV9392 (MH643816) and the second as an absence of 31 bp in ITV9395 (MW007723). All five genomes presented 22.9% of GC content, with 13 protein-coding genes, 22 tRNA genes, two rRNA (12S and 16S). The only observed divergence in the coding regions of the analyzed mitogenomes occurred probably due a non-synonymous transition from G to A in the second base of the codon for the sixth amino acid residue of the CYTB gene in ITV986 (MW007724), causing a change from arginine to glutamine. Most genes were encoded in the L-strand, while NAD1, NAD4, NAD4L, NAD5, eight tRNA genes and the two rRNA genes were in the H-strand. All ORFs presented a methionine-coding start codon, with five starting with ATG, five with ATT and three with ATA.

In the phylogenetic analyses, both ML and BI approaches resulted in similar tree topologies, differing only in the statistical support of the clades, with some nodes presenting low bootstrap values (BS < 70) (Figure 1). The BI analysis resulted in most clades with the maximum values of posterior probabilities (PP = 1), except for the node grouping *Agabus uliginosus* and *Colymbetes fuscus*, although still with a good support value (PP = 0.96) (Figure 1). On the other hand, some of the nodes within Hydroporinae presented low bootstrap support values in the ML tree. In addition, only four out of the 11 described subfamilies of Dytiscidae had representatives with available mitogenomes (Figure 1). As observed in previous phylogenetic approaches (Ribera et al. 2008; Michat et al. 2017), the tribe Hydroporini was recovered here as polyphyletic, with the group formed by the species of *Hydroporus* as sister to the remaining species of the family, while *Paroster* spp. appeared as sister to the clade formed by the representatives of the tribes Bidessini and Hyphydrini (Figure 1). *Desmopachria* sp. was recovered as monophyletic with maximum statistical support (PP = 1; BS = 100), appearing as sister to *Limbodessus palmulaoides*, but only distantly related and supported only in the BI analysis (PP = 1) (Figure 1).

**Figure 1.**
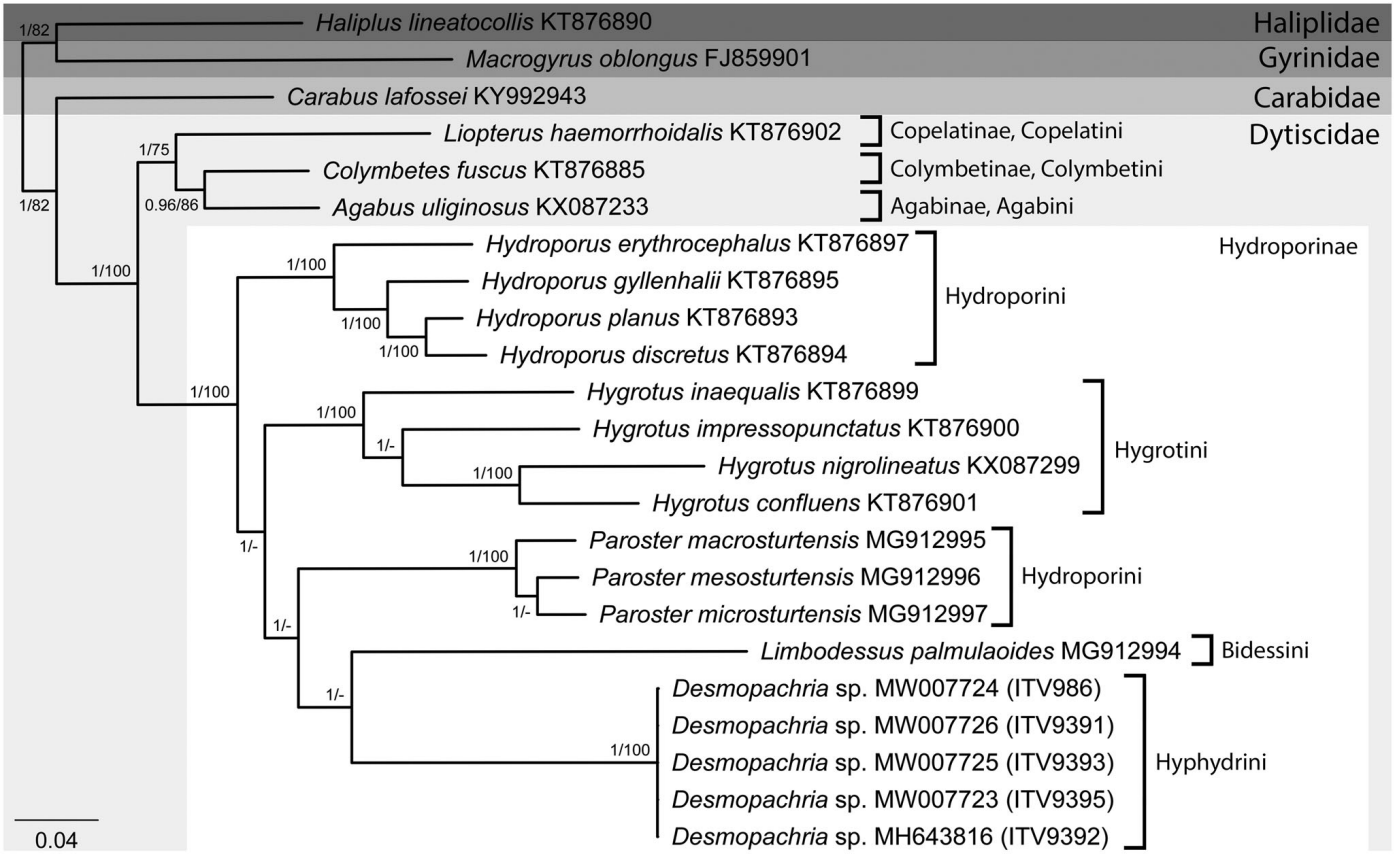
Majority-rule consensus phylogram of the Bayesian inference showing the phylogenetic relationships among our sampled specimens of *Desmopachria* sp. (ITV986, ITV9391, ITV9392, ITV9393 and ITV9395) and all the other species of Dytiscidae with available complete mitochondrial genomes, plus species of the families Carabidae, Gyrinidae and Haliplidae as outgroups, with the respective GenBank accession numbers. Subfamily and tribe affiliations are showed besides the brackets and statistical support values of the Bayesian inference (posterior probabilities) and maximum likelihood (bootstrap values above 70) approaches, respectively, are evidenced near the nodes of the clades.

## Data Availability

The data that support the findings of this study are available in NCBI at https://www.ncbi.nlm.nih.gov, reference numbers MH643816, MW007723, MW007724, MW007725 and MW007726.
